# Evidence of intrapulmonary penetration of nacubactam, a novel β-lactamase inhibitor, following coadministration of nacubactam with meropenem in healthy adults

**DOI:** 10.1128/aac.00026-26

**Published:** 2026-04-30

**Authors:** Jun Morita, Kazuya Ishiwata, Shogo Matsumoto, Seiji Kato, Gemma Attley, Katie Patel, Ignacio Rodriguez, Navita L. Mallalieu

**Affiliations:** 1 Meiji Seika Pharma Co., Ltd., Tokyo, Japan; 2 Roche Pharma Research and Early Development, Roche Innovation Centre New York, Little Falls, New York, USA; 3 Global Product Development, Roche Products Limited, Welwyn Garden City, United Kingdom; 4 Roche Pharma Research and Early Development, Roche Innovation Centre New York, New York, New York, USA; University of Houston, Houston, Texas, USA

**Keywords:** β-lactamase inhibitor, meropenem, nacubactam, pharmacokinetics, intrapulmonary penetration

## Abstract

Nacubactam is a novel non-β-lactam β-lactamase inhibitor being developed in combination with β-lactam antibiotics to treat serious gram-negative bacterial infections including nosocomial pneumonia. This study evaluated the systemic and intrapulmonary pharmacokinetics and safety of nacubactam plus meropenem in healthy adult male and female volunteers. Post-treatment bronchoalveolar lavage (BAL) to collect lung epithelial lining fluid (ELF). A total of 21 healthy adult participants were enrolled to receive a single dose of nacubactam 2 g coadministered with meropenem 2 g, of whom 20 underwent BAL and collection of blood samples for plasma pharmacokinetic (PK) characterization. The ratios of ELF to plasma area under the concentration-time curve from 0 to 8 h were 0.271 for nacubactam and 0.183 for meropenem. The estimated nacubactam half-life in ELF was 1.82 h and that in plasma was 1.71 h. PK data for meropenem were aligned with previously published results, with lower concentrations in ELF compared with plasma. The intrapulmonary penetration of nacubactam was favorable, with an area under the curve ratio (epithelial lining fluid to plasma) of 0.271, which was comparable to or greater than that observed for other approved β-lactamase inhibitors. The treatment combination was well tolerated, and no significant safety signals were identified. These data support further consideration of nacubactam/β-lactam combinations for the treatment of serious nosocomial pneumonia.

**CLINICAL TRIALS:**

This study was registered with ClinicalTrials.gov as NCT03182504.

## INTRODUCTION

Ventilator-associated pneumonia (VAP) and hospital-acquired pneumonia (HAP) are associated with significant morbidity and mortality among critically ill patients (1–3) and contribute to high healthcare costs (4–6). The microbial etiology varies, but the most frequently isolated pathogens are gram-negative bacteria (7, 8). Increasingly, these causative bacteria are multi-drug resistant (MDR), extensively drug resistant, or even pan-drug resistant, making them challenging to treat with currently available antibiotics (9). Infections with carbapenem-resistant Enterobacteriaceae are a particular concern due to the limited availability of effective and safe treatment options (10, 11).

Nacubactam (OP0595, RO7079901) is a novel non-β-lactam β-lactamase inhibitor being developed in combination with β-lactam antibiotics such as meropenem to treat serious gram-negative bacterial infections (12, 13). Nacubactam belongs to the diazabicyclooctane class of compounds and exhibits a dual mechanism of action: direct inhibition of penicillin-binding protein 2 (PBP2) in Enterobacteriaceae and inhibition of serine β-lactamases, including classes A and C and some class D enzymes (14). Thus, nacubactam exerts direct antibacterial effects, plus indirect effects via the protection of partner β-lactam antibiotics from degradation (15, 16).

In order to successfully treat VAP and HAP, nacubactam must be able to achieve adequate exposures in lung alveoli (17). The physicochemical properties of the compound and preclinical data obtained in mice indicated that nacubactam is able to distribute into the lung epithelial lining fluid (ELF) (18, 19). This study was conducted to confirm and characterize the intrapulmonary penetration of nacubactam in healthy volunteers using post-treatment bronchoalveolar lavage (BAL) to collect lung aspirate. As nacubactam is intended to be coadministered with β-lactam antibiotics such as meropenem, the intrapulmonary penetration of meropenem was also explored as a secondary study objective, along with the safety and tolerability profile of the drug combination.

## RESULTS

### Study population

**Fig 1:**
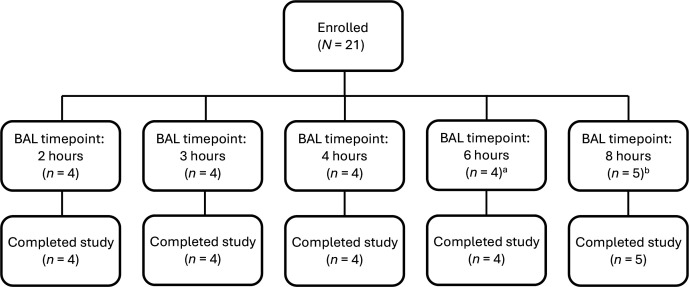
Number of participants at each BAL timepoint. Subject disposition is shown. ^a^Includes one participant in whom ELF was not calculable due to urea being below the limit of detection. ^b^Includes one participant who did not undergo the BAL procedure. BAL, bronchoalveolar lavage; ELF, epithelial lining fluid.

A total of 21 healthy adult participants received the study drugs (i.e., nacubactam 2 g and meropenem 2 g) and were included in the pharmacokinetic (PK) and safety analyses. One participant did not undergo a BAL procedure due to the onset of a moderate severity migraine and was replaced. The replacement participant was assigned to the same BAL sampling timepoint (Fig. 1).

Nacubactam and meropenem ELF total concentration data were available from 19 participants; the participant without BAL had no ELF concentration data, and one participant had a plasma urea concentration on Day 1 that was below the limit of quantification (BLQ), which precluded derivation of concentrations from BAL aspirate. Baseline characteristics are shown in Table 1.

**TABLE 1:** Baseline characteristics of healthy adults enrolled in the study^*a*^

	Nacubactam/meropenem (*N* = 21)
Age (years)	
Mean (SD)	37.9 (10.2)
Median (range)	37.0 (24–57)
Female sex, *n* (%)	11 (52.4)
Race, *n* (%)	
White	20 (95.2)
African American	1 (4.8)
BMI (kg/m^2^)	
Mean (SD)	25.9 (2.4)
Temperature (°C)	
Mean (SD)	36.7 (0.2)
BAL timepoint (h)	
2	4 (19.0)
3	4 (19.0)
4	4 (19.0)
6	4 (19.0)
8	5 (23.8)

^*a*^ BAL, bronchoalveolar lavage; SD, standard deviation.

### Pharmacokinetics

The protein binding rate of nacubactam (unpublished company records) is negligible (the unbound fraction is nearly 1). Therefore, the total concentrations are used for all subsequent calculations.

**Fig 2:**
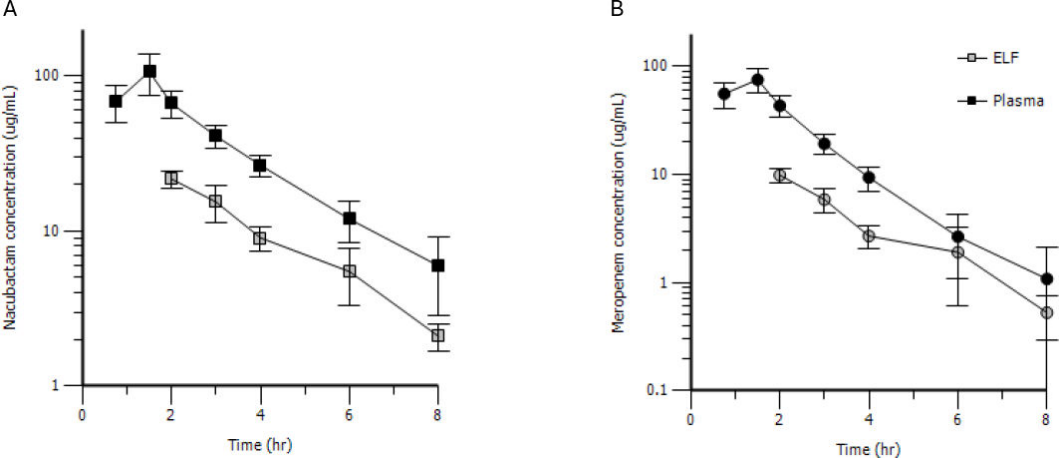
Mean profile of plasma and ELF concentration. Mean (±SD) plasma concentration-time profiles of nacubactam (**A**) and meropenem (**B**) after a 1.5-h IV infusion dosing with 2 g nacubactam coadministered with 2 g meropenem (log scale, all study participants [*N* = 21]). ELF, epithelial lining fluid; SD, standard deviation.

Nacubactam concentrations in ELF were 0.330–0.481 of the corresponding plasma exposures at each timepoint (Fig. 2A). Meropenem concentrations in ELF were also lower than the corresponding plasma exposures at each timepoint (Fig. 2B).

Although ELF concentrations could not be calculated for one participant because plasma urea concentrations were BLQ, both nacubactam and meropenem concentrations in BAL aspirates from this individual were similar to corresponding values from the other three participants who underwent the BAL procedure at the same timepoint (6 h).

Nacubactam concentrations in ELF appeared to be in rapid equilibrium with plasma concentrations, as ELF:plasma ratios were stable over the collection period with values of 0.342 at 2 h, 0.367 at 3 h, and 0.330 at 4 h post-dose (Table 2). The estimated nacubactam half-lives in ELF and plasma were similar (*t*_1/2_ = 1.82 and 1.71 h, respectively). Meropenem concentrations in ELF and plasma were not in instantaneous equilibrium. The estimated meropenem half-life in ELF was higher than that in plasma (*t*_1/2_ = 1.57 and 1.11 h, respectively). One participant was found to be an outlier, with an unexpectedly high volume of distribution of both nacubactam (44.1 L vs 15.2 L in the overall PK population) and meropenem (77.9 L vs 16.5 L in the PK population) despite having an estimated clearance (nacubactam, 8.6 L/h; meropenem, 18.8 L/h) slightly higher than that of other participants (nacubactam, 6.9 ± 1.2 L/h; meropenem, 12.4 ± 2.2 L/h, *N* = 21). Consequently, peak plasma concentrations were low, and *t*_1/2_ was prolonged for both nacubactam and meropenem. At the time of BAL collection (3 h), plasma concentrations in this individual (nacubactam, 39.5 µg/mL; meropenem, 17.9 µg/mL) were within the range seen in other participants (nacubactam, 31.4–57.6 µg/mL; meropenem, 13.0–25.7 µg/mL), and the ELF concentrations and ELF:plasma ratios were not markedly different from the other three participants who underwent the BAL procedure at the same timepoint. No explanation could be found for this discrepancy and, thus, all data were included in the study analyses.

**TABLE 2:** Summary of pharmacokinetic parameters after dosing of 2 g nacubactam coadministered with 2 g meropenem^*b*^^,^^*c*^^,^^*d*^

Parameters	Nacubactam		Meropenem	
	Plasma (*n* = 21)	ELF (*n* = 19)^*a*^	Plasma (*n* = 21)	ELF (*n* = 19)^*a*^
CL (L/h)	6.85 (17)	NA	12.4 (18)	NA
*V*_ss_ (L)	15.2 (35)	NA	16.5 (43)	NA
AUC_0–8h_ (µg·h/mL)	275 (19)	74.6 (NA)	159 (21)	29.1 (NA)
AUC_inf_ (µg·h/mL)	292 (17)	80.1 (NA)	161 (18)	30.3 (NA)
*C*_max_ (µg/mL)	102 (34)	21.6 (13)	73.0 (34)	9.8 (14)
*T*_max_ (h)	1.5 (1.5–2.0)	2.0 (NA)	1.5 (1.5–2.0)	2.0 (NA)
*C*_last_ (µg/mL)	5.32 (51)	2.1 (21)	0.8 (78)	0.5 (48)
*t*_1/2_ (h)	1.71 (22)	1.82 (NA)	1.11 (23)	1.57 (NA)

^*a*^ ELF was not calculable for two study participants: one (in the 6 h group) had urea below the limit of detection, and one (in the 8 h group) did not undergo the BAL procedure.

^*b*^ Data are shown as geometric mean (coefficient of variation %), except *T*_max_, which is shown as median (range).

^*c*^ AUC_0–8h_, area under the concentration-time curve from 0 to 8 h (relative to the start of dosing); AUC_inf_, area under the concentration-time curve from 0 h to infinity; BAL, bronchoalveolar lavage; CL, clearance; *C*_last_, last observed concentration; *C*_max_, maximum observed concentration; ELF, epithelial lining fluid; NA, not applicable; *T*_max_, time of maximum plasma concentration; *t*_1/2_, terminal half-life; *V*_ss_, volume of distribution at steady state.

^*d*^ This change is a bit confusing; suggest leaving it as original: ELF:plasma ratios. If you wish to add the AUC ratio, then modify the title as follows: Summary of ELF:plasma ratios presented as concentration or AUC ratios after dosing of 2 g nacubactam coadministered with 2 g meropenem, or leave the title as original and add a footnote to “ratios” and to indicate “ratios presented as concentration or AUC ratio”.

ELF:plasma ratios, measured as concentration and area under the curve (AUC) ratios at each timepoint are summarized in Table 3. The nacubactam and meropenem ELF:plasma AUC ratios were 27% and 18%.

**TABLE 3:** Summary of ELF:plasma ratios after dosing of 2 g nacubactam coadministered with 2 g meropenem^*c*^^,^^*d*^

Time (h)	*n*	Nacubactam ELF:plasma ratio	Meropenem ELF:plasma ratio
AUC_0–8h_	19	0.271	0.183
2	4	0.342 (0.063)	0.229 (0.016)
3	4	0.367 (0.059)	0.281 (0.064)
4	4	0.330 (0.032)	0.273 (0.070)
6	3^*a*^	0.481 (0.098)	0.900 (0.522)
8	4^*b*^	0.452 (0.184)	0.799 (0.584)

^*a*^ Samples were obtained for four participants, but ELF could not be calculated for one individual due to urea being below the limit of detection.

^*b*^ Five participants were allocated to this timepoint, but one did not undergo the BAL procedure.

^*c*^ Data are shown as arithmetic means (standard deviation).

^*d*^ BAL, bronchoalveolar lavage; ELF, epithelial lining fluid.

### Safety

At least one adverse event (AE) was experienced by six (28.6%) participants (Table 4). Most AEs were graded as mild; two were moderate; and none were severe in intensity. There were no deaths or serious AEs, and all AEs resolved without sequelae. No participants discontinued due to AEs.

**TABLE 4:** Summary of adverse events^*b*^

	Nacubactam/meropenem (*N* = 21)
Subjects with any adverse event	6 (28.6)
Total number of adverse events	9^*a*^
Severe adverse event	0
Serious adverse event	0
Deaths	0
Withdrawals due to adverse event	0
Adverse events (preferred term)	
Presyncope	3 (14.3)
Cough	1 (4.8)
Diarrhea	1 (4.8)
Dysgeusia	1 (4.8)
Migraine	1 (4.8)
Nausea	1 (4.8)
Physical assault/facial injury	1 (4.8)^*a*^

^*a*^ For one participant, two events (physical assault and facial injury) were reported in two different Medical Dictionary for Regulatory Activities categories (injury, poisoning and procedural complications, and social circumstances) in the output but were considered here as a single adverse event.

^*b*^ Data are shown as *n* (%).

The most frequently reported AE was presyncope (reported as “vasovagal reaction”) in three (14.3%) participants. No other AE was reported in more than one participant during the study. Among the nine AEs reported, three (diarrhea, migraine, and nausea) were considered by the investigator to be related to the combination of nacubactam and meropenem.

There were no clinically relevant abnormalities in laboratory test results, electrocardiogram (ECG) parameters, or vital signs, or any related AEs reported during the study.

## DISCUSSION

Antimicrobial resistance is commonly referred to as a “silent pandemic” due to the significant threat it poses to global health (20). The spread of carbapenem-resistant gram-negative bacteria has made treatment increasingly challenging and resulted in elevated mortality for infected patients (21–23). Several β-lactam/β-lactamase combinations have been approved by regulatory authorities in recent years for use against MDR pathogens (24), and yet more combinations are undergoing clinical testing (25). However, while the introduction of new antibiotic combinations to treat HAP and VAP has widened the choice of empiric treatment, additional agents and combinations with broad-spectrum activity and low toxicity are still needed to combat MDR infections (26).

When administered alongside β-lactam antibiotics, such as meropenem, nacubactam is active against bacteria producing any of the four molecular classes of β-lactamases, including metallo-β-lactamases (class B) and oxacillinase (class D) (14–16). Nacubactam has demonstrated potent *in vivo* therapeutic effects (27, 28), suggesting that nacubactam/β-lactamase combinations may have applicability in treating a range of bacterial infections. However, understanding the PK and pharmacodynamic pulmonary properties of antibiotics is critical to selecting the optimal treatment regimen (29), and in order for the combination of nacubactam and meropenem to be successful in the treatment of HAP and VAP, their ability to achieve adequate pulmonary exposure must be demonstrated.

Previous non-clinical data in rodent models have indicated that nacubactam is able to distribute into pulmonary ELF (18, 19), while meropenem has been shown to be present in ELF at 63% of those in plasma (30). In this study, nacubactam concentrations in ELF were approximately one-third of the corresponding plasma exposures and appeared to be in rapid equilibrium with plasma. This was consistent with published data indicating that β-lactams, carbapenems, and β-lactam inhibitors all have limited lung penetration (31). Meropenem ELF:plasma ratios in this study were slightly lower (48%) compared with those in a previous study investigating meropenem-vaborbactam (63%) (30). However, they were more closely aligned with a study evaluating meropenem alone, which did not have sufficient information to calculate an ELF:plasma ratio for a 2 g dose, but reported ratios of 43% for a 500 mg 1.5-h intravenous (IV) infusion and 28% for a 1 g 1.5-h IV infusion (32). Although differences in study designs and calculation methodology preclude direct comparisons, both previously published studies and the current analysis found that meropenem concentrations in ELF are not in instantaneous equilibrium with plasma concentrations, and the ELF:plasma ratio changes across the sampling interval. Table 3 shows higher variability in meropenem ELF:plasma ratios at later vs earlier timepoints. This difference could be due to a combination of individual physiological differences and the pharmacokinetic properties of meropenem in this study. High variability in ELF:plasma ratios at 6 and 8 h post-dose was primarily attributed to a significant plasma PK outlier (one subject, high *V*_ss_) causing terminal phase fluctuations, which were directly amplified in pooled ELF data by the single-point BAL sampling design. Nacubactam exhibited favorable intrapulmonary penetration (27% [by AUC ratio] or 39% [by concentration ratio]), comparable to or exceeding other approved β-lactamase inhibitors. This high penetration, confirmed by preclinical PK/PD models demonstrating significant bacterial kill, translated to robust ELF concentrations that consistently exceeded target MICs, thereby facilitating attainment of the PK/PD driver and supporting nacubactam’s potential efficacy in gram-negative pneumonia.

The treatment combination was well tolerated, and no significant safety signals were identified. Although presyncope was reported in 14% of participants in this study, these events were mild/moderate, reversible, and considered not related to the study drug; in addition, this type of AE was not reported in previously published Phase 1 nacubactam studies (13). Furthermore, it has long been known that vasovagal responses are relatively common in people who undergo cannula insertion for IV infusion (33). A more detailed analysis of the safety profile of nacubactam in a larger population will be possible when the results of the Integral-1 trial (study ID: jRCT2031230075) of nacubactam antibiotic combination in patients with complicated urinary tract infections or acute uncomplicated pyelonephritis are published.

The ELF data from this study indicate that nacubactam is likely to achieve sufficient lung exposure. These results, alongside the good safety and tolerability profile reported to date, support further development of nacubactam for the treatment of patients with pneumonia.

The strengths of this study include that the design was similar to that previously used to assess other antibiotics (30, 34) and allowed direct comparison with historical meropenem data. The use of ELF drug concentrations in healthy volunteers as a surrogate of intrapulmonary drug penetration in patients with pneumonia is considered to be a robust investigational technique (17), with no differences observed in antibiotic lung penetration between healthy volunteers and pneumonia patient populations (31). Although each participant in our study only underwent a single BAL procedure, the pooling of individual data allowed derivation of ELF concentration vs time profiles for the population, while collection of corresponding plasma concentration data ensured that each participant had their own internal control data for PK modeling of the kinetics of distribution into the intrapulmonary space.

Limitations using a healthy population without acute or chronic illnesses may not reflect critically ill patients with pulmonary dysfunction and potential extrapulmonary complications. Furthermore, this study included only a single IV dose of treatment, whereas real-world management of infection may require repeated dosing over multiple days, which could result in higher frequency or severity of AEs.

In conclusion, the results from this study of the intrapulmonary penetration of nacubactam coadministered with meropenem support further consideration of this combination as a potential treatment for patients with serious nosocomial pneumonia.

## MATERIALS AND METHODS

### Study design

This was a Phase 1, non-randomized, open-label, one-treatment, one-group study to investigate the intrapulmonary lung penetration of nacubactam in healthy adult male and female volunteers. The study was conducted at a single investigational site in the United States between 2 June 2017 and 10 August 2017.

The study consisted of a screening visit (Days −28 to −1), a treatment and sampling period (Day 1), and a safety follow-up visit (Days 7–14). On Day 1, participants received a single 1.5-h IV infusion of 2 g nacubactam coadministered with 2 g meropenem. The two drugs were administered concurrently as separate solutions through two separate lines via a large bore, double-lumen catheter, under the supervision of study personnel.

This was followed by blood sampling for PK testing and a single BAL procedure to collect lung ELF for measurement of intrapulmonary concentrations of nacubactam and meropenem. While all participants received the same study drug treatment and followed the same blood sampling scheme, each individual was assigned to one of five possible timepoints (2, 3, 4, 6, or 8 h after the start of study drug administration) for the BAL procedure. Equal numbers of participants were assigned to each timepoint, and assignment of individual participants was at the discretion of the investigator.

### Study population

Eligible participants were adults aged 18–60 years, with a body mass index between 18 and 30 kg/m^2^, and who had been non-smokers for at least 6 months prior to screening. Subjects were medically healthy, without evidence of active or clinically significant chronic disease(s) identified from a detailed medical and surgical history, physical examination (including vital signs and 12-lead ECG), and laboratory safety test results. Female participants were required to have a negative pregnancy test result, and all participants agreed to use an effective method of contraception during the study. All eligible participants provided written informed consent for study participation.

During screening, participants were excluded if they had a history of asthma or clinically significant lung disease; any condition which would contraindicate study treatment or procedures; a history of any clinically significant medical condition or recent change in health status; positive test for human immunodeficiency virus, hepatitis B, or hepatitis C; recent history of addiction or positive test for alcohol or drugs of abuse; or donation or loss of >500 mL of blood within the three months before screening.

### Blood sample collection

Blood samples to measure plasma nacubactam and meropenem concentrations were collected at 0.75, 1.5, 2, 3, 4, 6, and 8 h after study treatment administration on Day 1. An additional plasma sample was taken at the same time as part of the BAL procedure to measure urea. The plasma samples were stored at −80°C pending analysis.

### BAL collection

Subjects were assigned to one of five BAL timepoints (2, 3, 4, 6, or 8 h after study treatment administration). Participants underwent a BAL procedure for collection of BAL samples, as mentioned below: a fiberoptic bronchoscope was passed into the right middle lobe of the lung after appropriate topical anesthesia (lidocaine). Sterile saline solution (50 mL, 0.9%) was introduced into the lung and then immediately aspirated. This aspirate was discarded, and the aspirate trap was changed. The process was repeated with three further 50 mL volumes of sterile saline, and the volume of each aspirate was measured. The second, third, and fourth aspirates were pooled, mixed, and then centrifuged to separate alveolar macrophages from the supernatant. Aliquots of supernatant were removed and frozen prior to shipment to the bioanalytical laboratory for measurement of nacubactam, meropenem, and urea concentrations. The BAL samples were stored at −80°C without addition of stabilizers such as MOPS.

### Bioanalytical methods for plasma and BAL

Nacubactam, meropenem, and urea concentrations in plasma and BAL samples were measured using validated bioanalytical assays. Urea was assessed because, when conducting drug concentration calculations in ELF, BAL data can be combined with measurement of urea to overcome potential bias due to sample dilution (35, 36).

#### Drug concentration assays

Covance Laboratories Ltd. (Harrogate, UK) conducted the bioanalytical assays to determine the concentrations of nacubactam and meropenem in both plasma and BAL fluid.

#### Urea assays

Keystone Bioanalytical Inc. (North Wales, PA, USA) was responsible for measuring urea concentrations in plasma and BAL fluid, which were used as a dilution marker to calculate ELF concentrations.

The QC and BLQ information for plasma and urea concentration measurements of nacubactam and meropenem met requirements for precision and accuracy and is shown in Table 5.

**TABLE 5:** Bioanalytical specifications for plasma and urea concentration of nacubactam, meropenem, and urea

Sample	Matrix	BLQ^*a*^ (µg/mL)	Precision (RSD%)			Accuracy (%)
			LQC^*b*^	MQC^*c*^	HQC^*d*^	
Nacubactam	Plasma	1	7.2	5.2	4.5	98.6–104.3
	BAL	0.001	2.5	5.6	4.2	98.3–100.8
Meropenem	Plasma	0.1	10.8	3.0	2.6	98.0–100.3
	BAL	0.001	11.2	7.4	6.6	94.3–102.4
Urea	Plasma	100	0.9–1.3	0.4	1.1	−7.6% to 1.7% (as %RE)
	BAL	0.2	8.0	1.0	2.5	−1.6% to 4.3% (as %RE)

^*a*^ BLQ, below the limit of quantification.

^*b*^ LQC, low quality control.

^*c*^ MQC, middle quality control.

^*d*^ HQC, high quality control.

#### Data analysis

##### ELF concentration calculations

Concentrations of nacubactam and meropenem in ELF were derived from drug concentrations in BAL aspirate samples according to published methodology (37). In summary, the concentration of urea in the BAL fluid was compared to the concentration of urea in the plasma (37). This standard method accounts for the dilution of ELF by the saline used during the BAL procedure.


$$C_{ELF}=C_{BAL}\times Urea_{Plasma}/Urea_{BAL},$$

where *C*_BAL_ is the drug concentration measured in the BAL fluid.

##### Pharmacokinetic calculations

PK parameters were estimated by non-compartmental analysis using Phoenix WinNonlin (version 6.4; Certara USA, Inc.) by Covance.

### Safety assessments

Available preclinical and clinical data did not indicate a requirement for specific safety monitoring measures, with no anticipated acute safety risks associated with a single 2 g IV infusion dose of nacubactam coadministered with 2 g of meropenem in healthy adults. Safety assessments included physical examination, monitoring of vital signs, ECG, laboratory assessments (hematology, blood chemistry, and urinalysis), and AEs.

### Statistical analysis

The sample size was determined based on feasibility and no statistical power calculations were conducted. In alignment with similar studies performed for other investigational drugs, it was anticipated that 20 participants would be adequate for characterization of nacubactam and meropenem pharmacokinetics in ELF (i.e., four per BAL timepoint). Replacement in the event of withdrawal was at the discretion of the investigator and study sponsor.

Concentration data were summarized using descriptive statistics and graphical presentations; there was no inferential statistical analysis of any PK parameter. For analyses of ELF data, all available concentration data were pooled, and arithmetic mean concentrations at each nominal timepoint were derived. Safety data were also summarized descriptively. AEs were categorized using Preferred Terms from the Medical Dictionary for Regulatory Activities (version 20.0).

## Data Availability

The data sets generated during and/or analyzed during the current study are available from the corresponding author upon reasonable request.
